# 10-channel phased-array coil for carotid wall MRI at 3T

**DOI:** 10.1371/journal.pone.0288529

**Published:** 2023-08-09

**Authors:** Matthijs H. S. de Buck, Peter Jezzard, Robert Frost, Chris Randell, Katherine Hurst, Robin P. Choudhury, Matthew D. Robson, Luca Biasiolli

**Affiliations:** 1 Wellcome Centre for Integrative Neuroimaging, FMRIB Division, Nuffield Department of Clinical Neurosciences, University of Oxford, Oxford, United Kingdom; 2 Athinoula A. Martinos Center for Biomedical Imaging, Massachusetts General Hospital, Charlestown, MA, United States of America; 3 Department of Radiology, Harvard Medical School, Boston, MA, United States of America; 4 PulseTeq Limited, Chobham, United Kingdom; 5 Nuffield Department of Surgical Sciences, University of Oxford, Oxford, United Kingdom; 6 Acute Vascular Imaging Centre, Division of Cardiovascular Medicine, Radcliffe Department of Medicine, University of Oxford, Oxford, United Kingdom; 7 Oxford Centre for Clinical Magnetic Resonance Research, Division of Cardiovascular Medicine, Radcliffe Department of Medicine, University of Oxford, Oxford, United Kingdom; 8 Perspectum, Gemini One, John Smith Drive, Oxford, United Kingdom; German Cancer Research Center: Deutsches Krebsforschungszentrum, GERMANY

## Abstract

**Background:**

Accurate assessment of plaque accumulation near the carotid bifurcation is important for the effective prevention and treatment of stroke. However, vessel and plaque delineation using MRI can be limited by low contrast-to-noise ratio (CNR) and long acquisition times. In this work, a 10-channel phased-array receive coil design for bilateral imaging of the carotid bifurcation using 3T MRI is proposed.

**Methods:**

The proposed 10-channel receive coil was compared to a commercial 4-channel receive coil configuration using data acquired from phantoms and healthy volunteers (N = 9). The relative performance of the coils was assessed, by comparing signal-to-noise ratio (SNR), noise correlation, g-factor noise amplification, and the CNR between vessel wall and lumen using black-blood sequences. Patient data were acquired from 12 atherosclerotic carotid artery disease patients.

**Results:**

The 10-channel coil consistently provided substantially increased SNR in phantoms (+77 ± 27%) and improved CNR in healthy carotid arteries (+62 ± 11%), or reduced g-factor noise amplification. Patient data showed excellent delineation of atherosclerotic plaque along the length of the carotid bifurcation using the 10-channel coil.

**Conclusions:**

The proposed 10-channel coil design allows for improved visualization of the carotid arteries and the carotid bifurcation and increased parallel imaging acceleration factors relative to a commercial 4-channel coil design.

## 1. Introduction

Atherosclerosis in the carotid arteries is one of the leading causes of stroke [1–4], with the majority of plaque accumulation occurring near the carotid bifurcation. Accurate assessment of the size, shape, location, and composition of atherosclerotic plaques [1, 3, 5] is important for the effective diagnosis and treatment of the disease and the prevention of ischemic events.

Magnetic resonance imaging (MRI) can be used for non-invasive *in vivo* characterization of atherosclerotic plaque in the carotid arteries [5–7]. The different contrast weightings in MRI facilitate a comprehensive characterization of the vessel wall and the plaque [8–11], as well as visualization of the arterial blood flow. Accurate MRI assessment of plaque size and composition, which are indicative of plaque vulnerability [2, 12], is constrained by the carotid image resolution and signal-to-noise ratio (SNR) that can be achieved within a clinically reasonable scan time.

Moreover, plaque lipid content can be accurately quantified by T2 mapping on a voxel-by-voxel basis, as has been demonstrated in endarterectomy patients by histological validation [7, 11, 13, 14]. This MRI technique can be used to study the relationship between plaque lipid content and symptomatic status, and to identify patients at higher risk of plaque rupture. However, it requires sufficient SNR in multiple spin echo images at different echo times to generate robust T2 estimates for each plaque voxel, thus it would clearly benefit from increased coil sensitivity at the carotid depth.

The carotid bifurcation is located in a relatively superficial part of the neck, at a typical depth of 3 cm below the skin [15–17], albeit deeper in overweight patients, who are at higher risk of atherosclerotic complications. Both the longitudinal location (here longitudinal is defined as the location along the vessel in the head-foot direction) and the depth of the carotid bifurcation can vary substantially among subjects due to anatomical differences in the neck and the vasculature. This means that an effective MR receive coil for imaging near the carotid bifurcation requires high SNR at a sufficiently large penetration depth and longitudinal coverage to accommodate a wide range of anatomies.

In addition to high SNR, accurate carotid plaque characterization requires high-resolution images to accurately visualize the detailed (<0.5 mm) features of the plaque composition. Parallel imaging techniques [18] are often used to acquire data at high resolutions with reduced scan times, at the cost of a loss in SNR. The relative loss of SNR can further degrade depending on the coil geometry being used [19], so coil configurations which provide low amounts of noise amplification at high acceleration factors are desirable for carotid MRI [20, 21].

The advantages of phased-array coils for carotid artery imaging have been established by Hayes et al. [22]. For imaging near the carotid bifurcation at 3T, which is recommended over 1.5T in clinical practice because of its increased SNR [10] and high clinical availability, various studies into the optimal coil configurations are available [15, 17, 20, 23–26]. Those studies at 3T use between 4 and 16 coil channels for bilateral imaging. A 30-channel coil for carotid MRI has been shown to facilitate high parallel imaging acceleration factors, but with limited SNR penetration [20].

Coil configurations consisting of few but large receive elements typically benefit from large spatial coverage, but with limited SNR [17, 20]. Increased numbers of small coil channels can provide improved superficial SNR, but with reduced penetration depth and flexibility [20, 26]. In this work, a new 10-channel coil configuration for accurate bilateral visualization of the carotid bifurcation using parallel imaging acceleration is proposed. The achieved SNR, noise amplification, and vessel visualization using this coil are compared to the results obtained using a commercial 4-channel carotid coil for phantom and *in vivo* acquisitions. In a recent paper, Zhang et al. [20] compare the performance of three different (6-, 8-, and 30-channel) carotid coil designs to the performance of the same commercial 4-channel coil which is used in this study. Therefore, the performance of the 10-channel coil proposed here relative to the 4-channel coil is compared to their results to put the performance of the 10-channel coil into the context of those other designs.

## 2. Methods

### 2.1 Coil design

**Fig 1:**
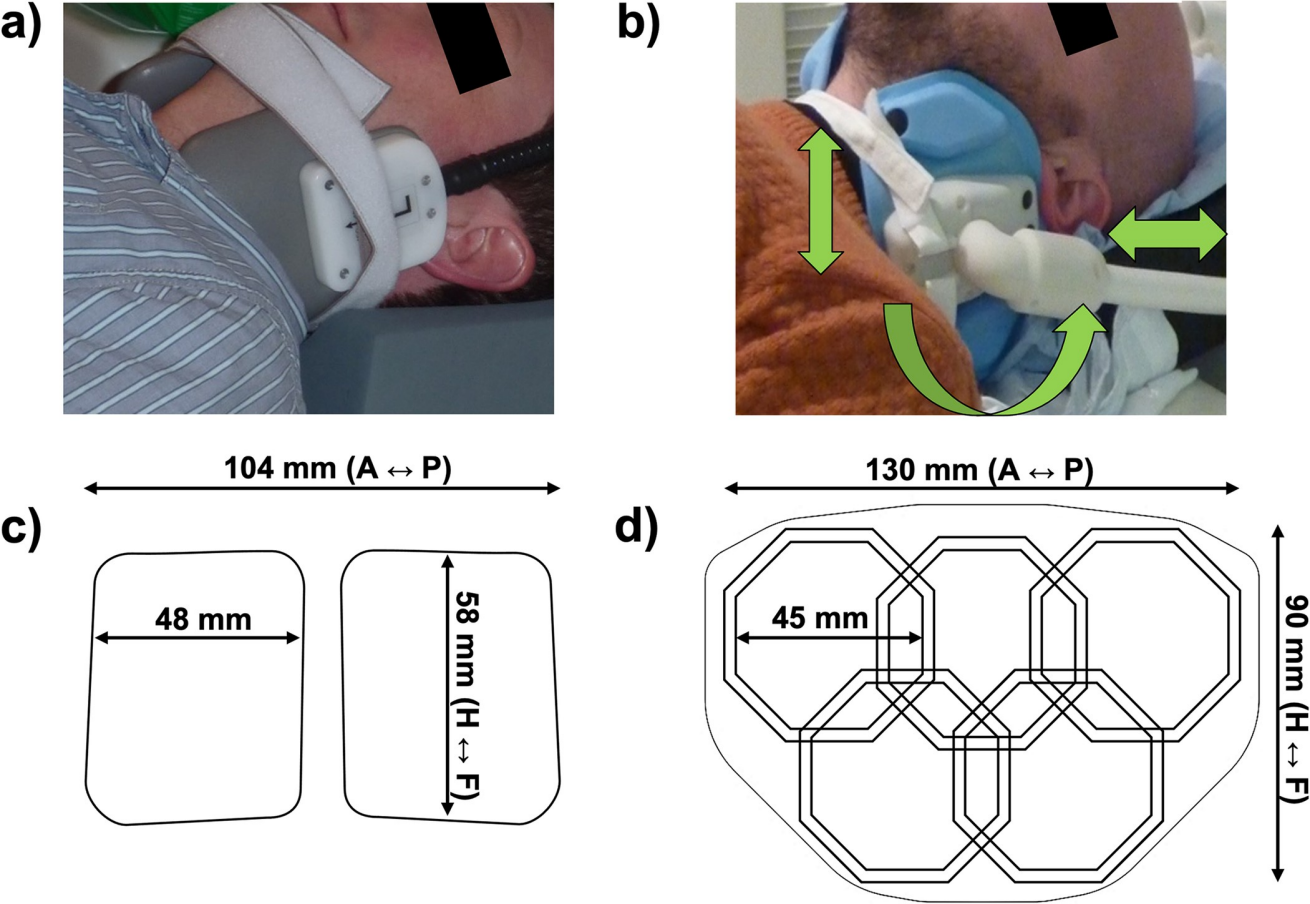
The two coils used in this work. **(a)**: The 4-channel coil positioned around the neck of a volunteer. **(b):** The 10-channel coil positioned around the neck of a (different) volunteer. The green arrows indicate the degrees of freedom of the coil positioning around the neck of the subject. **(c-d)** Relative positions and dimensions of 4-channel [20] and 10-channel coils and their individual channels (figures show one of the bilateral sides).

All data were acquired using a newly developed 10-channel phased-array receive coil (PulseTeq, Chobham, United Kingdom) and are compared to results obtained from a widely used commercial 4-channel phased-array receive coil (MachNet BV, Roden, The Netherlands). The measurement setups using both coils are shown in Fig 1. Both coils were designed for bilateral imaging of the carotid arteries near the carotid bifurcation.

The 4-channel coil consists of two bilateral sets of paired transverse channels. The 10-channel surface coil consists of two sets of octagonal elements with custom-made low-impedance preamplifiers, positioned in an ‘Olympic ring’-configuration (Fig 1D) which allows for the coil to be wrapped around the neck without compromising patient comfort. The 10-channel coil design was based on preliminary measurements of the carotid depth and distance from the jawline in 44 patients (age = 67.8 ± 11.9 years, 33 males). The average depth of the carotid bifurcation center lines was 32 ± 7 mm, with a minimum of 18 mm and a maximum of 48 mm (consistent with [15–17]). Each side of the coil (overall dimension = 130 × 90 mm^2^) is made of 5 elements of size = 45 mm with both active and passive protection. The coils were designed to be flexible and adaptable to different neck sizes and are mounted on support arms that can bend and rotate for improved positioning with respect to the carotid bifurcation while maintaining high patient comfort. The coils were surrounded by foam covers to ensure optimal patient comfort and adequate isolation. Two hook-and-loop straps were used to fixate the coils around the neck.

### 2.2 Phantom study

Data from a cylindrical short T1 phantom (15 cm diameter) were acquired for quantitative comparison of the g-factor noise amplification performance of the two coils. Multi-slice T1-weighted turbo-spin-echo data were acquired for estimation of phantom g-factor maps of both coils in both coronal (10 slices) and transverse (20 slices) scan orientations. Sequence parameters include TR/TE = 1000 ms/13 ms, resolution 0.9 × 0.9 × 2.0 mm^3^, in-plane matrix size 256×256, 100% slice gap, and turbo factor 10. The total scan time was 1:21 and 1:50 minutes for the coronal and transverse orientations, respectively.

### 2.3 *In vivo* study

Nine healthy volunteers (mean ± SD in age and weight: 33.2 ± 7.0 years; 78 ± 5 kg) were imaged with both the 4- and 10-channel coils on a Siemens (Erlangen, Germany) Verio 3T scanner. To estimate T2 maps of the arterial wall in the healthy volunteers, 5 transverse slices over 10 cm centered at the carotid bifurcation were acquired using the DANTE-MESE sequence [11, 27]. The following Multi-Echo Spin-Echo (MESE) acquisition parameters were used: 14 echoes (TE = 9.1 to 127.4 ms), TR = 2000 ms, FOV = 128 × 128 × 100 mm^3^, matrix size = 192 × 192, voxel size 0.67 × 0.67 × 2 mm^3^, slice gap = 100%, scan time 4 min. A Delay Alternating with Nutation for Tailored Excitation (DANTE [28]) preparation before each readout was used for flowing blood signal suppression. The following DANTE parameters were used: gradient amplitude = 18 mT/m, 120 RF pulses, flip angle = 8°, RF pulse interval = 500 μs. Bright-blood Time-of-Flight (TOF) MR Angiography (MRA) data were acquired to localize the carotid bifurcations.

Additional multi-slice T1-weighted turbo-spin-echo data were acquired in one healthy volunteer for the estimation of *in vivo* SNR and g-factor maps. 12 transverse slices were acquired with TR/TE = 1090 ms/13.1 ms, resolution 0.6 × 0.6 × 2.0 mm^3^, in-plane matrix size 256 × 252, 100% slice gap, turbo factor 7, and total scan time 2:41 minutes.

Healthy volunteer data were acquired under an agreed technical development protocol approved by the Oxford University Clinical Trials and Research Governance office (SOP OHBA_009_V1), in accordance with International Electrotechnical Commission and UK Health Protection Agency guidelines. The authors had no access to additional information that could identify individual participants after data collection.

12 patients with atherosclerosis (72.3 ± 9.4 years, 80.6 ± 11.7 kg) were scanned at carotid plaque locations using slices perpendicular to the direction of the vessel with the 10-channel coil using DANTE-FSE (Fast Spin Echo) T1-weighted imaging. FSE acquisition parameters were TR = 1280 ms, TE = 13 ms, FOV = 150 × 150 mm^2^, matrix size 256 × 256 (0.59 × 0.59 mm^2^ resolution), echo train length = 7, slice thickness = 2 mm, slice gap = 100%, and scan time 3 min. DANTE preparation parameters were gradient amplitude = 18 mT/m, 64 RF pulses, flip angle = 8°, and RF pulse interval = 1 ms. Bright-blood TOF MRA data were acquired to localize carotid bifurcations and lumen stenoses. 13 interleaved T1-weighted slices were acquired at the level of the atherosclerotic plaques (affected carotid side based on Doppler Ultrasound). Ethical approval for patient scans was obtained from the UK National Research Ethics Services (reference 15/NW/0972) and patients provided written informed consent. Patient data were acquired between 2016 and 2019.

### 2.4 Image analysis

In the phantom and healthy volunteer studies, data were reconstructed using MATLAB R2019a (Mathworks, Natick, MA). Channel-by-channel noise correlation matrices were calculated from noise-only measurements consisting of 512 samples for each coil.

For the reconstruction of T1w turbo-spin-echo data, the Berkeley Advanced Reconstruction Toolbox (BART; v0.4.02) [29, 30] was first used for ESPIRiT receive coil sensitivity estimation [31]. Then, separately acquired noise-only measurements were used for reconstruction in B1-weighted SNR units [32, 33]. For fully-sampled acquisitions without parallel imaging acceleration, those were calculated as

$$SNR=\sqrt{2}\frac{\left|S^{H}N^{-1}I_{ch}\right|}{\sqrt{S^{H}N^{-1}S}},$$ (1)

where *SNR* denotes the B1-weighted reconstructed image with pixel intensities in SNR units, calculated from the ESPIRiT coil sensitivities *S*, the bandwidth-scaled noise correlation matrix *N*, and the multi-coil image data *I_ch_*. Superscript *H* denotes the conjugate transpose operation. For comparison of the SNR in vessel wall segments, a semi-automated gradient detection algorithm [34] was used for vessel wall delineation. This algorithm (git.fmrib.ox.ac.uk/ndcn0873/acutance_TdB) provides signal and contrast information about the vessel wall along 90 radial directions (at 4° intervals) from the center of the vessel. This boundary delineation method was previously found to achieve excellent inter- and intra-observer agreement, as well as high reproducibility of results on repeated measurements of the same vessel from different scan sessions [34]. The latter makes this a useful approach for the quantitative comparison of data acquired from the same volunteers in separate scan sessions (using the two different coils). For SNR comparison, the maximum vessel wall SNR along each of the 90 radial directions was compared on reconstructions from both coils.

The geometry factor (g-factor) noise amplification metric is used to assess the parallel imaging performance of a receiver coil when using methods such as SENSE [19] or GRAPPA [35]. For an acceleration factor of R, the reconstructed SNR (*SNR*_*PI*_) is given by

$$SNR_{PI}=\frac{SNR}{g\sqrt{R}}.$$ (2)

The spatially variant g‐factor noise amplification of both coils was estimated for the (phantom and *in vivo*) turbo-spin-echo acquisitions at retrospectively undersampled acceleration factors of 2 and 3. For this, the method described by Breuer et al. [36] was used to calculate the g-factors after application of different GRAPPA kernels, using a calibration region of 32×32 k-space points.

Wall/lumen contrast-to-noise ratio (CNR) for the healthy volunteers was estimated as the SNR difference between the carotid vessel wall and its lumen. Inner and outer vessel wall boundaries were again segmented following published procedures [11, 34]. Resulting CNR values were compared for images acquired using the 10-channel and the 4-channel coil. For all nine volunteers, results were compared at each of the 14 different echo times.

Since images were acquired with the 10-channel and 4-channel coil at different times (scan-rescan during the same session), identical voxel locations could not be assumed for quantitative statistical analysis. Therefore, we tested the null hypothesis that data were independent random samples drawn from the same normal distribution, using a two‐sample t-test at a 5% significance level.

Voxel-wise T2 values were estimated in the carotid wall by fitting an exponential decay curve to the signal intensity of the 14 echoes using a Levenberg-Marquardt nonlinear least squares algorithm [11]. T2 maps were generated using data acquired with both the 10-channel and the 4-channel coil. Statistical comparisons were performed for the estimated T2 values of the healthy vessel wall and their standard errors using a two‐sample t-test.

All MATLAB code used for reconstruction and analysis is shared online (DOI: 10.5287/ora-gae410mwj). In addition to code, this also includes all phantom data and (reconstructed) healthy volunteer data, as well as the segments of the reconstructed patient data underlying the results presented in this paper.

## 3. Results

### 3.1 Signal-to-noise ratio

**Fig 2:**
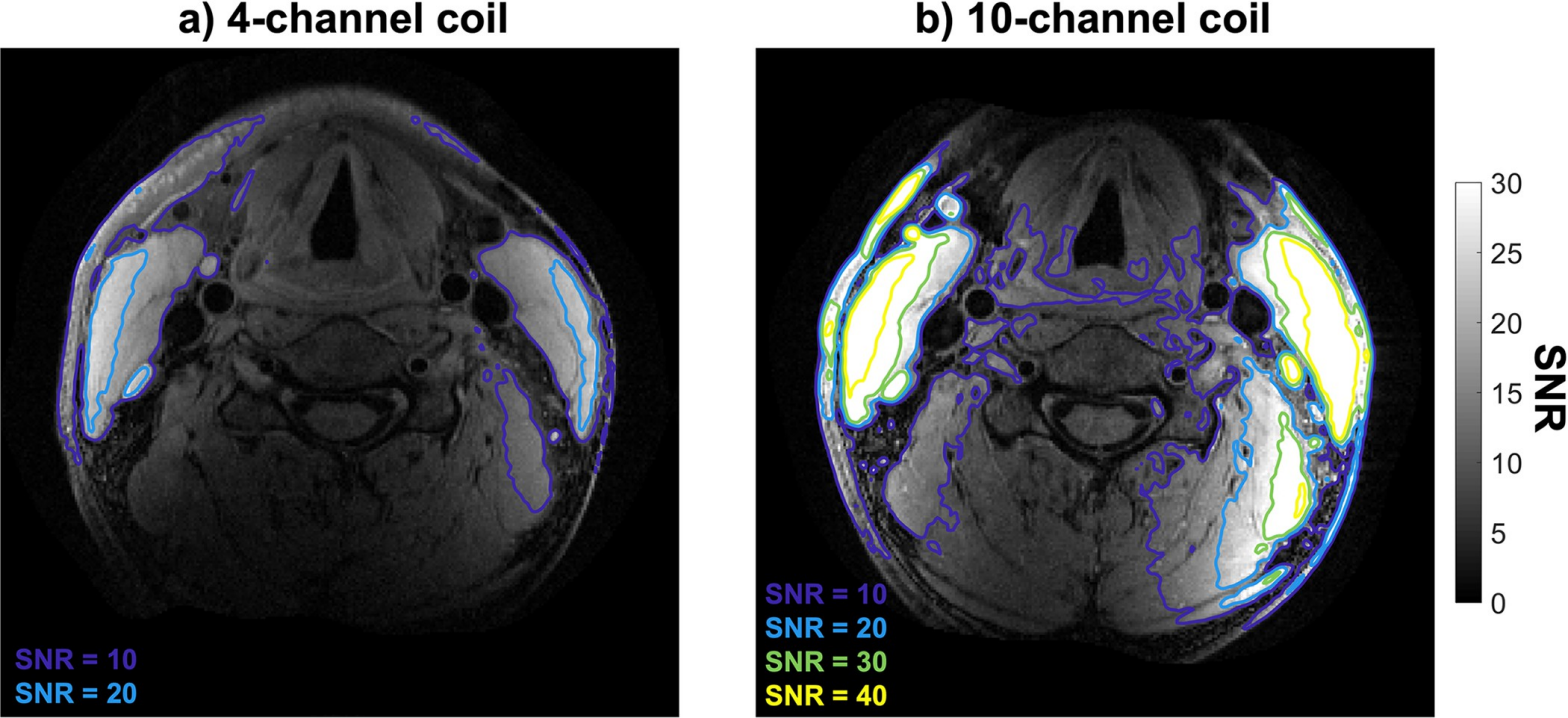
SNR-units reconstructions from transverse T1-weighted turbo-spin-echo data for one healthy volunteer SNR, acquired without parallel imaging acceleration using (a) the 4-channel coil and (b) the 10-channel coil. SNR contours are shown for both acquisitions at iso-contour levels of 10, 20, 30, and 40.

Reconstructions of T1-weighted turbo-spin-echo acquisitions in SNR units from a healthy volunteer using both the 4-channel coil and the 10-channel coil are shown in Fig 2. The 10-channel coil consistently provides an increased SNR relative to the 4-channel coil, with higher increases closer to the bilateral sides of the neck in the left-right direction: the SNR = 20 iso-contour line for the 4-channel coil is observed in an anatomically similar area at the side of the neck to the SNR = 40 iso-contour line for the 10-channel coil. Although both coils show a distinct drop in SNR further from the coil elements, the 10-channel coil maintains a higher SNR level throughout the transverse slice. The mean (± standard deviation) SNR gain across the 90 radial directions for the carotid artery segments in the acquisitions in Fig 2 is 77 ± 27%, increasing the overall average vessel wall SNR from 7.2 for the 4-channel coil to 12.6 for the 10-channel coil (p < 0.001).

### 3.2 Noise correlation

**Fig 3:**
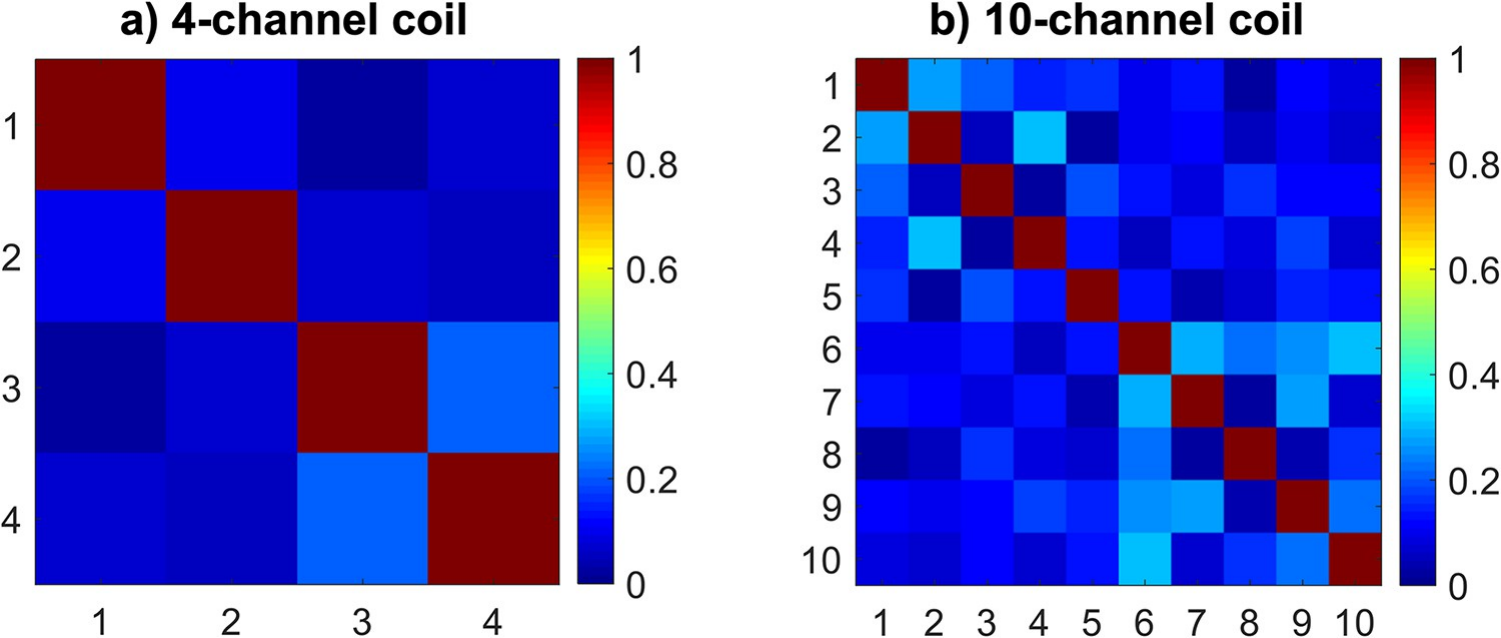
Noise correlation matrices for (a) the 4-channel coil and (b) the 10-channel coil. The unilateral coil elements correspond to indices 1–2 and 3–4 for the 4-channel coil and indices 1–5 and 6–10 for the 10-channel coil.

Fig 3 shows noise correlation matrices for both coils. The average magnitude of the off-diagonal components is 0.09 ± 0.06 for the 4-channel coil and 0.13 ± 0.08 for the 10-channel coil (unilateral average off-diagonal magnitudes are 0.15 ± 0.06 and 0.17 ± 0.10, respectively). The maximum off-diagonal values are 0.20 for the 4-channel coil (between channels 3 and 4) and 0.31 for the 10-channel coil (between channels 2 and 4).

### 3.3 g-factor noise amplification

**Fig 4:**
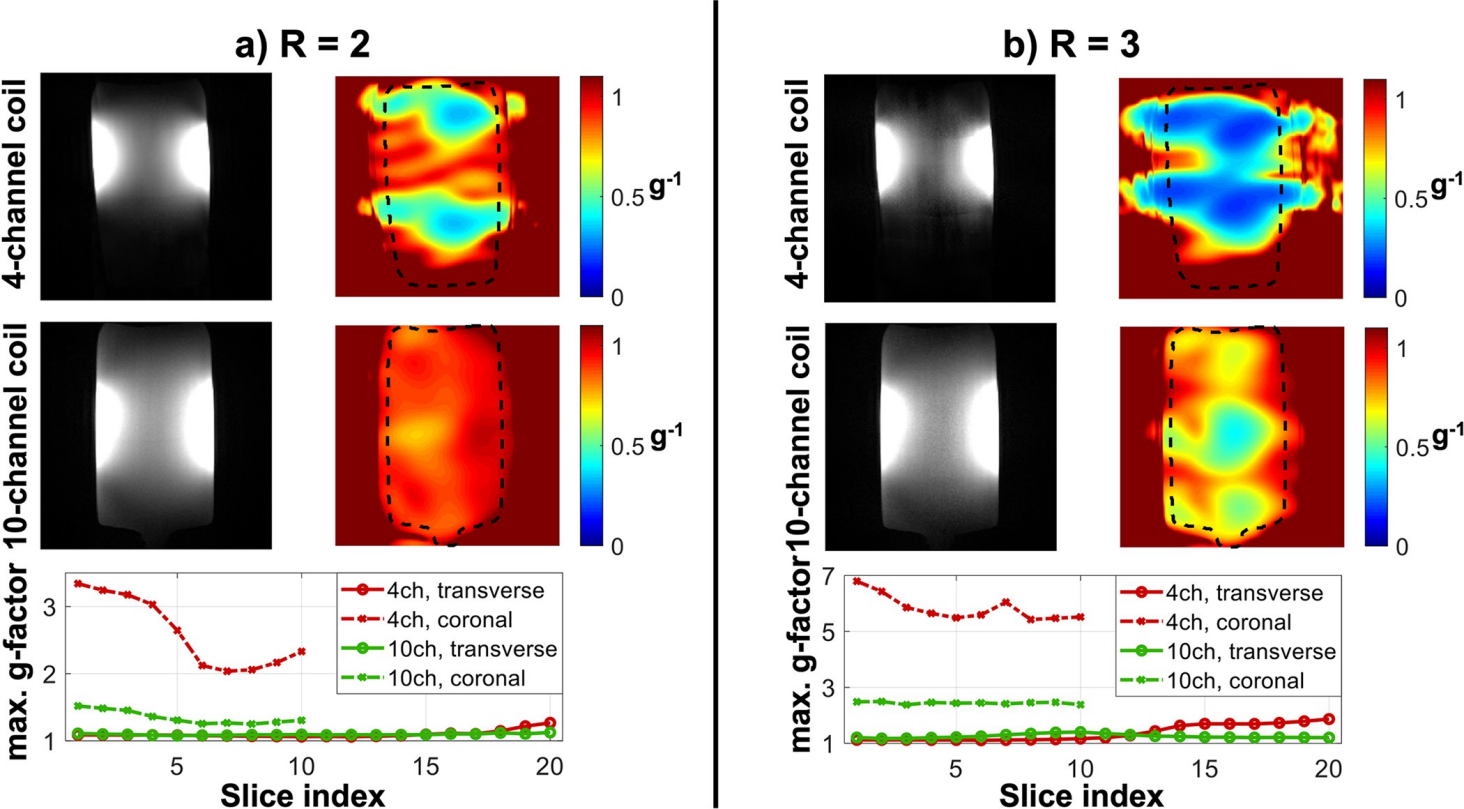
Estimated g-factor noise amplification in a phantom using the 4-channel and the 10-channel coils, at (a) R = 2 and (b) R = 3. Reconstructions as well as retained SNR (inverse g-factor) maps of a single coronal slice are shown for both coils and at both acceleration factors. The bottom row shows the maximum g-factor in each slice for both the transverse and coronal acquisitions. Note that the maximum g-factor values are shown using a different y-axis scaling in Fig (a) than in Fig (b).

Fig 4 shows the estimated g-factor noise amplification in the phantom using both coils at R = 2 (Fig 4A) and R = 3 (Fig 4B). The two top rows show examples of the reconstructed slices and g-factor distributions in the phantom in a single coronal slice for all four cases (both coils and both GRAPPA acceleration factors). The bottom row shows the maximum noise amplification values of all coronal and transverse slices. For coronal acquisitions, the 10-channel coil consistently achieves a significant (p < 0.001) g-factor reduction of 47 ± 7% at R = 2, and of 58 ± 3% at R = 3. For transverse acquisitions, the noise amplification is lower for both coils, probably due to the combination of smaller in-plane size of the phantom and increased spatial separation of receive channels in transverse acquisitions. Lower g-factors are visible for the 10-channel coil for some of the off-center transverse slices where the 4-channel coil has an increased maximum g-factor. However, no overall statistically significant difference is observed between the two coils for the transverse acquisitions.

**Fig 5:**
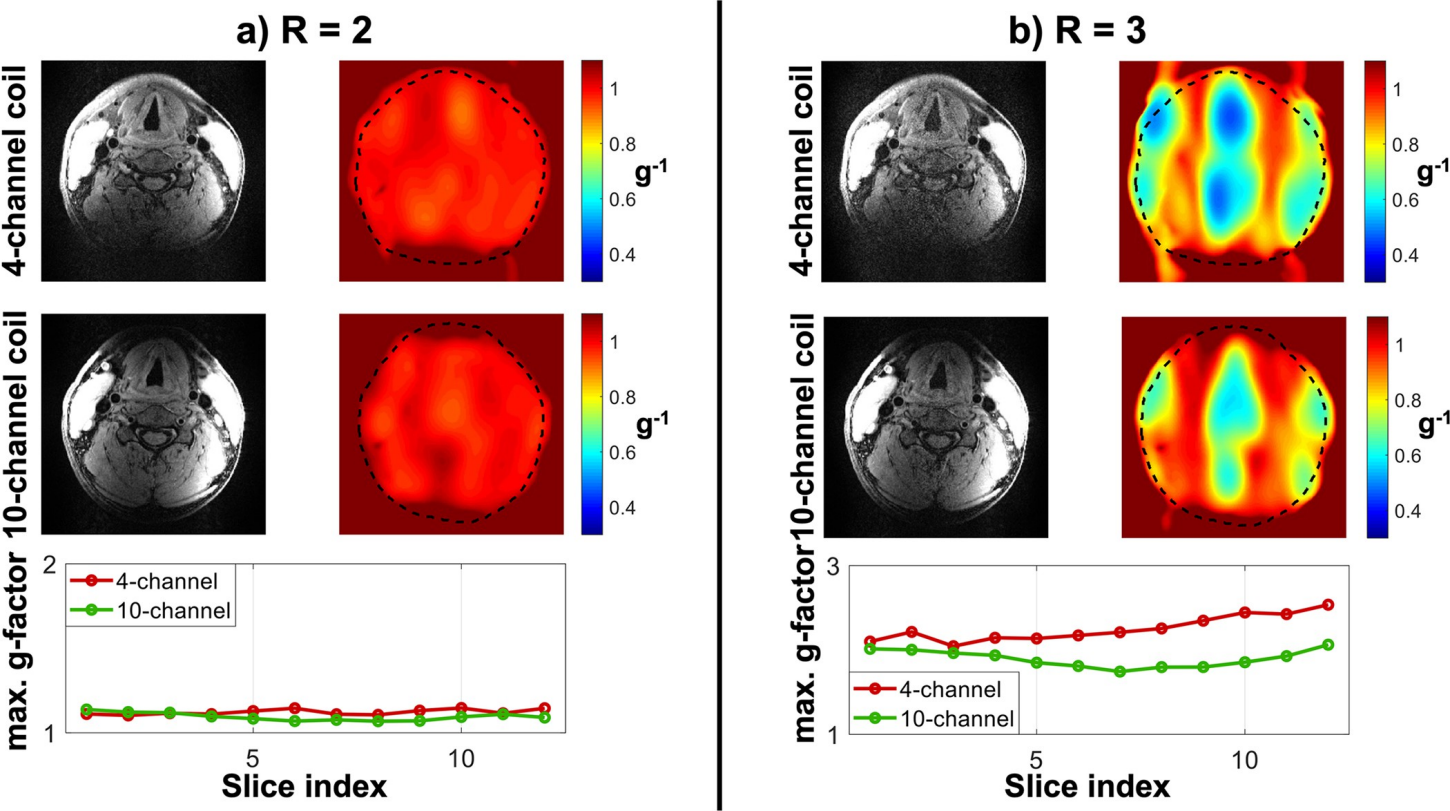
*In vivo* estimated g-factor noise amplification using the 4-channel and the 10-channel coils, at (a) R = 2 and (b) R = 3. All data is shown for transverse acquisitions. The top two rows show reconstructions as well as retained SNR (inverse g-factor) maps of a single transverse slice for both coils and at both acceleration factors. The bottom row shows the maximum transverse g-factor in each slice. Note that the maximum g-factor values are shown using a different y-axis scaling in Fig (a) than in Fig (b).

Fig 5 shows the estimated *in vivo* g-factor noise amplification using both coils at R = 2 (Fig 5A) and R = 3 (Fig 5B) for transverse acquisitions (reconstructed from the same data as the SNR maps in Fig 2). The 10-channel coil achieves a small but significant (p = 0.003) g-factor reduction of 3 ± 3% at R = 2, and of 19 ± 9% at R = 3 (p < 0.001).

### 3.4 CNR and T2 quantification

**Fig 6:**
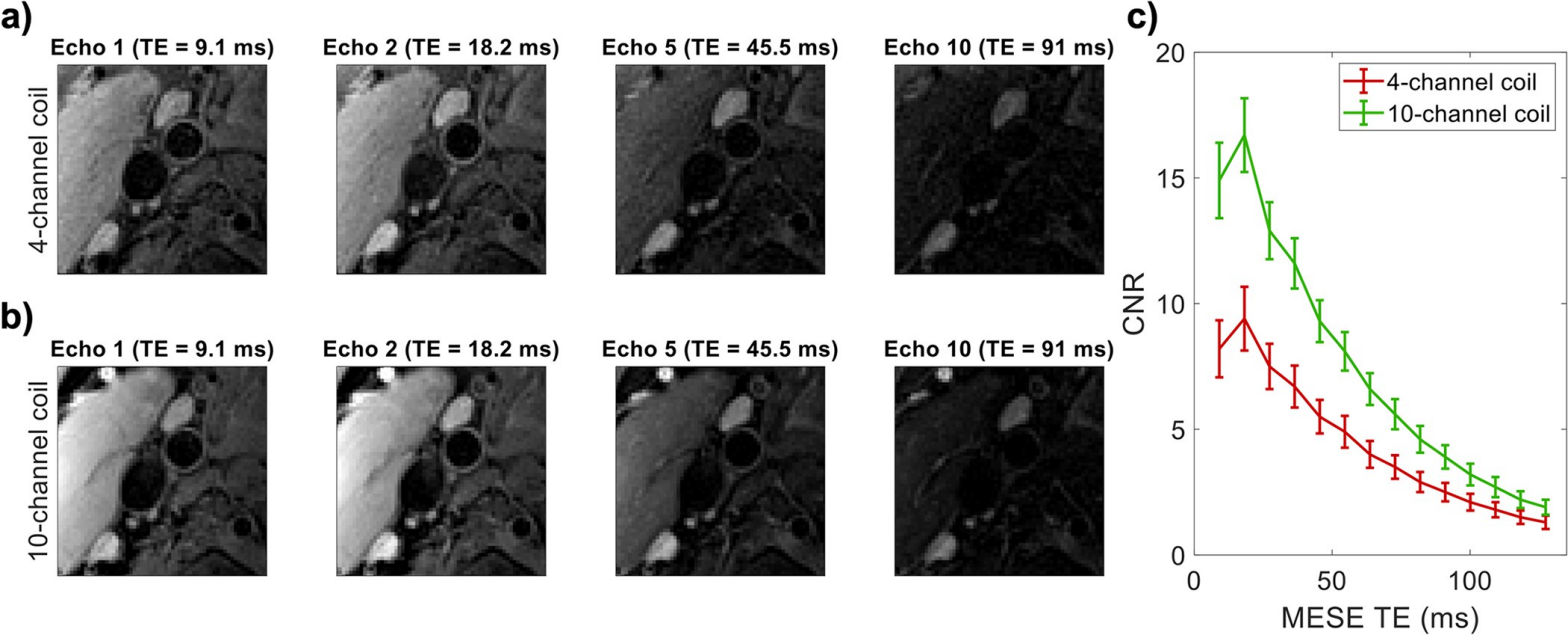
DANTE-MESE scans of nine healthy volunteers using both coils. All data is shown to the same greyscale. **(a-b)** Close-ups near the carotid bifurcation of a single volunteer at four different echo times using **(a)** the 4-channel coil and **(b)** the 10-channel coil. **(c)** Mean carotid wall/lumen CNR results of both coils across the nine volunteers.

DANTE-MESE images at different echo times are shown in Fig 6 for both the 4-channel and the 10-channel carotid coil. At short echo times, the internal and external carotid arteries are clearly visible on the images acquired from both coils. At longer echo times, the increased CNR when using the 10-channel coil versus the 4-channel coil noticeably improves vessel visibility. The mean CNR between the vessel walls and the lumen is shown in Fig 6C for all subjects using both coils. The CNR is consistently significantly higher (+62 ± 11% for the 14 echo times; *p*<10^−5^ at each individual echo time) when using the 10-channel coil, with the largest relative increases (up to +82%) at short echo times. In total, ~14,000 vessel wall voxels were identified and compared across the 9 healthy volunteers.

The T2 value of healthy carotid wall tissue at 3T calculated for the 9 volunteers using the 14 echo times was 65.9±14.1 *ms* (mean + SD) using the 4-channel coil, and 63.1±13.6 *ms* using the 10-channel coil. The standard error of the T2 estimates was 6.6±4.5 *ms* using the 4-channel coil, and 5.0±3.3 *ms* using the 10-channel coil. The statistical distributions of data acquired with the 4-channel coil and 10-channel coil were significantly different for T2 values and standard errors (all tests rejected the null hypothesis with *p*<10^−5^). The improved SNR obtained with the 10-channel coil resulted in an average reduction of 24% on the standard errors of the estimated T2 values.

### 3.5 Patient validation

**Fig 7:**
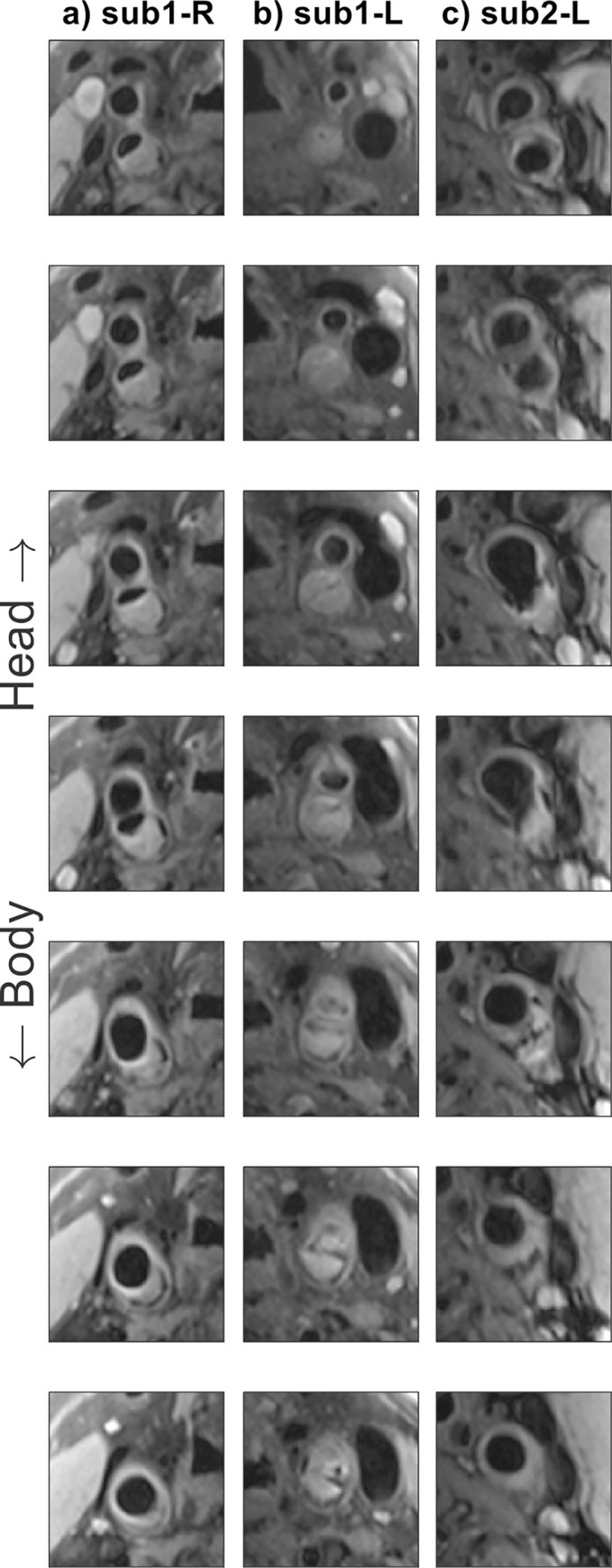
Consecutive DANTE-FSE T1-weighted slice segments showing the carotid bifurcations in two patients with atherosclerotic carotid artery disease, acquired using the new 10-channel coil. **(a-b)** Subject 1, left- and right-hand sides; **(c)** subject 2, right-hand side.

Fig 7 shows typical examples of DANTE-FSE T1-weighted images of the carotid arteries near the carotid bifurcation in two patients with atherosclerotic carotid artery disease, acquired using the 10-channel coil.

## 4. Discussion

In this study, a 10-channel phased-array coil design for carotid imaging at 3 Tesla was compared to a commercial 4-channel coil design. Data acquired in a phantom and in healthy volunteers were used to compare the SNR, noise correlation, parallel imaging noise amplification, vessel wall-lumen CNR, and derived quantitative T2 values using both coils. Additional patient data were included to show the typical image quality and carotid plaque details that can be achieved by the proposed coil.

The healthy volunteer data in Fig 2 show a significant increase in SNR (77 ± 27% along the carotid walls) when using the proposed 10-channel coil compared to the commercial 4-channel coil. The largest increase in SNR was observed close to the coils, but a consistent SNR increase was present throughout the phantom. In patients, the large increase in SNR near the edge of the neck is beneficial for imaging the relatively superficial carotid bifurcations. The consistent SNR improvement at greater depths indicates that a benefit can be maintained for patients with thicker necks or with atypical vasculature. Since the longitudinal position of the carotid bifurcation can vary by several centimeters between patients, the observed longitudinal consistency makes the SNR gain in the 10-channel coil beneficial to large groups of patients without requiring adjustments in coil positioning during a scan session. The SNR maps in Fig 2 show a slight left-right asymmetry for both coils, mainly in the posterior half of the neck. This may be caused by slight asymmetry in coil placement but did not result in significant differences in the measured SNR in the carotid artery walls (closer to the anterior of the neck).

The channel-to-channel noise correlation in Fig 3 indicates similar performance between the two coils, but lower values for both the mean and the maximum off-diagonal correlation for the 4-channel coil. The observed results for the 10-channel coil are similar to the values reported for the 8-channel coil designs used by Hu et al. [23] and Zhang et al. [20]. While Zhang et al. [20] reported excellent decoupling (correlation of 0.03) between the unilateral elements of the same 4-channel coil design as the one used here, both our results and those presented by Hu et al. [23] for the same coil design found substantially higher unilateral coupling of around 0.15.

A reduction in parallel imaging noise amplification for coronal acquisitions in a phantom at both R = 2 and R = 3 is achieved when using the 10-channel coil (Fig 4). Although the phantom data did not obtain a statistically significant reduction in noise amplification for transverse acquisitions, the *in vivo* results using the 10-channel coil in Fig 5 did provide significantly reduced g-factors for transverse acquisitions at R = 3.

In 2016, Hu et al. [23] proposed an 8-channel carotid coil design, which they compared to the same commercial 4-channel coil design that is used for comparison in this paper. The 10-channel coil proposed here obtained a 77% SNR increase in the carotid arteries, consistently larger than the ~40% increase achieved in a phantom by Hu et al. [23]. This 77% SNR increase is also slightly larger than the 67% vessel wall SNR increase in T1-weighted acquisitions presented earlier by Balu et al. [15], who compared a different 8-channel carotid coil design to a custom-built 4-channel coil. The vessel wall SNR increase reported here is similar to the 73% SNR increase in bright-blood TOF-MRA data reported by Tate et al. [26] using a 16-channel coil design relative to the results using a 4-channel coil design. Due to the use of different image contrasts for SNR calculation (TOF-MRA versus black-blood vessel wall imaging) and the use of different 4-channel coil designs, comprehensive comparison of the performance of that 16-channel coil design and the 10-channel coil design proposed here requires further investigation. However, that 16-channel coil [26] requires a larger number of receive channels and provides reduced positioning flexibility. Finally, Zhang et al. [20] compared the vessel wall SNR in healthy volunteers using three different coil designs to the same 4-channel coil used here. For their 6- and 8-channel designs, they found significant increases in vessel wall SNR (using non-accelerated acquisitions) of around 35% and 26%, respectively. Their 30-channel coil design resulted in a significant decrease in vessel wall SNR, with the benefit of reduced image degradation at high parallel imaging acceleration factors.

Direct comparison of the SNR distributions presented in Fig 2 to the results found for 4-, 6-, 8-, and 30-channel coil designs by Zhang et al. [20] can provide further insight into the performance of the proposed coil relative to coils with various numbers of channels. For example, their 30-channel coil provided reduced SNR penetration into the neck, while their 6- and 8-channel coils provided similar SNR contour areas (equivalent to Fig 2 here) to the 4-channel coil. That is different from the results presented in Fig 2, which show a substantial increase in SNR penetration for the 10-channel coil relative to the 4-channel coil. However, it should be noted that despite using the same 4-channel coil design and also using *in vivo* data for SNR calculation, direct comparison between our and Zhang et al.’s results is limited by the use of different SNR calculation approaches, different sequences, unilaterally (instead of bilaterally) acquired data, and the observed differences in noise correlation.

The lower g-factors when using the 10-channel coil (Figs 4 and 5) make it possible to visualize the carotid arteries at increased parallel imaging acceleration factors with limited noise amplification. This is especially important for cases when high-resolution data are acquired, such as for volumetric plaque quantification, or when multiple datasets with different contrasts need to be acquired for tissue characterization. In such cases, acquisitions without additional acceleration would require prohibitively long scan times. In practice, the minimal g-factor noise amplification using the proposed 10-channel design at R = 2 means that data can be acquired with a substantial scan time reduction while maintaining clinical image quality. The large g-factor reduction in the longitudinal direction can be explained based on the difference in longitudinal position of some of the individual channels in the 10-channel coil, while the bilateral pairs of channels in the 4-channel coil are positioned in approximately the same longitudinal location.

The data acquired from healthy volunteers using the 10-channel coil and the 4-channel coil (Fig 6) show significant carotid wall-to-lumen CNR improvements using the 10-channel coil. The observed mean CNR increase of +62% (with up to +82% for the shortest TE) at the carotid bifurcation is consistently larger than that achieved by the 6-, 8-, and 30-channel coil designs as reported by Zhang et al. [20], who unilaterally measured CNR changes relative to the same 4-channel coil design used in this study, and with similar FOV and voxel size. Compared to the 4-channel coil, the increased CNR obtained using the 10-channel coil provided an improved vessel visibility around the carotid bifurcation at all echo times in the DANTE-MESE acquisitions, and a reduced error on the estimated T2 values of the healthy vessel wall across the 9 healthy volunteers.

In the patient data, as shown in Fig 7, the high CNR of the T1-weighted images provided by the 10-channel coil produced clearly visible and clinically useful vessel and plaque delineation over the length of the carotid bifurcation. The carotid bifurcation was clearly delineated for all 12 patients that were scanned using the 10-channel coil array, despite anatomical differences in longitudinal location of the bifurcations, benefiting from the increased longitudinal coverage of this proposed coil design. In compliance with the approved ethics agreement, additional patient data using the 4-channel coil were not acquired and are therefore not available for comparison in this study.

Overall, the 10-channel coil design proposed here achieved improved SNR, g-factor noise amplification, vessel-to-lumen CNR, and quantitative T2 estimates relative to the 4-channel coil. Comparisons of the SNR change reported here to similar results in literature indicate that the use of 10 channels can provide further increases in SNR magnitude and penetration relative to the 6- and 8-channel designs, while even higher numbers of coil channels can result in a reduction in SNR performance for carotid wall MRI (as was for example observed for the 30-channel coil design used by Zhang et al. [20]). The 10-channel coil configuration proposed in this study offers reduced g-factor noise amplification compared to the 4-channel coil, but further g-factor improvements have been reported when using coils with higher numbers of channels. This can be advantageous in specific scenarios where very high acceleration factors (R > 3) are required. However, for most clinical scenarios, the demonstrated SNR and CNR benefits of the proposed design can enable accurate imaging of the carotid bifurcation at high resolutions using multiple contrasts or quantitative mapping for plaque characterization within reduced scan times.

## 5. Conclusion

The proposed 10-channel phased-array coil configuration achieved better visualization of the carotid bifurcation compared to a commercial 4-channel coil design, allowing for improved carotid artery characterization within shorter scan times. Comparison to literature results indicates that the proposed design achieved increased SNR and CNR performance compared to 6-, 8-, and 30-channel coil designs.

## Abbreviations

CNR: contrast-to-noise Ratio
DANTE: delay alternating with nutation for tailored Excitation
FSE: fast spin echo
g-factor: geometry factor
GRAPPA: generalized autocalibrating partially parallel acquisition
MESE: multi-echo spin echo
MRA: magnetic resonance angiography
MRI: magnetic resonance imaging
RF: radiofrequency
SD: standard deviation
SENSE: sensitivity encoding
SNR: signal-to-noise ratio
TOF: = time-of-flight
TR/TE: repetition time/echo time
